# Reply to the Letter to the Editor: “Impact of mannitol on intracranial pressure assessed by optic nerve sheath ultrasonography during video-laparoscopic prostatectomy: a randomized clinical trial”

**DOI:** 10.1016/j.bjane.2026.844794

**Published:** 2026-07-16

**Authors:** George Pereira Barreto, Wallace Andrino da Silva

**Affiliations:** a Hospital Universitário Onofre Lopes, Division of Anesthesiology, Natal, RN, Brazil; b Universidade Federal do Rio Grande do Norte, Department of Surgery, Natal, RN, Brazil

*Dear Editor*,

We would like to thank Dr. Suresh and colleagues^1^ for their thoughtful comments regarding our recently published randomized clinical trial evaluating the impact of mannitol on intracranial pressure assessed by Optic Nerve Sheath Diameter (ONSD) ultrasonography during video-laparoscopic prostatectomy.^2^ We appreciate the opportunity to further discuss important methodological aspects related to bedside ONSD assessment.

We fully acknowledge that optic nerve sheath ultrasonography is an operator-dependent technique, and that observer-related variability is a well-recognized and widely discussed limitation of the method, particularly when interpreting submillimetric ONSD variations. Nevertheless, recent meta-analyses have demonstrated an adequate correlation between ONSD measurements and intracranial pressure, reinforcing the clinical applicability of this non-invasive bedside method.^3^

In our study, several methodological strategies were implemented to minimize measurement variability. All examinations were performed by anesthesiologists with more than five years of practical experience and specific training in optic nerve ultrasonography. In addition, ONSD measurements were obtained bilaterally at each predefined intraoperative time point, and the mean binocular value was used for statistical analysis. According to the study protocol, measurements would be repeated if the inter-eye difference exceeded 0.5 mm; however, this threshold was never reached, and no repeated measurements were required. Although formal intraobserver variability analysis was not performed, the standardized acquisition protocol and the absence of clinically significant inter-eye discrepancies suggest consistent image acquisition throughout the study. However, these measures do not replace formal assessment of intraobserver and interobserver reliability.

Regarding ultrasound mode limitations, we acknowledge that B-mode ultrasonography is susceptible to blooming-related artifacts and that ultrasound system settings may influence ONSD measurements. Although standardized A-mode ultrasonography has been proposed as a more accurate technique for delineating optic nerve sheath interfaces, it requires dedicated ophthalmic equipment and specific operator expertise, limiting its availability and applicability in most perioperative point-of-care settings, particularly in the operating room. Consequently, B-mode ultrasonography remains the technique most commonly used in perioperative and critical care research. Notably, the recent international consensus statement on ONSD point-of-care ultrasonography focused on standardizing B-mode image acquisition and measurement criteria and did not recommend the routine use of A-mode ultrasonography.^4^ We agree that future studies should report ultrasound acquisition settings in greater detail and continue efforts toward standardized imaging protocols to improve reproducibility.

The authors’ suggestion about storing frozen ultrasound images for subsequent blinded offline analysis is interesting and potentially valuable from a methodological perspective. However, this approach was not incorporated into our original study protocol. As ours is a teaching hospital with continuous development of perioperative ultrasonography protocols, we believe this may represent an important methodological refinement for future studies in this field.

Concerning the comments about age-related cerebral atrophy and possible anatomical changes in cerebrospinal fluid spaces in elderly patients, we agree that these factors should be considered when interpreting ONSD measurements. Previous studies have demonstrated that ONSD increases with age, particularly in individuals older than 65 years.^5^ However, in our study, the mean age was similar between groups (approximately 64 years in both groups), making it unlikely that age-related anatomical variations represented a major confounding factor in our analysis.

Another relevant aspect is the variation in ONSD according to ethnicity and population characteristics. Previous studies conducted worldwide have demonstrated substantial differences in normal ONSD values across populations, ranging from a mean of 3.68 mm (95% CI 2.85–4.40 mm) in a Canadian population^6^ to 5.1 mm (95% CI 4.7–5.4 mm) in a healthy Chinese population.^7^ These findings highlight the importance of establishing normative population-specific databases for more accurate interpretation of ONSD measurements in both screening and diagnostic settings.

Despite these recognized limitations and population-related variations in ONSD measurements, we would like to emphasize that the primary objective of our study was not to validate the ultrasonographic method itself, nor to compare imaging modalities for intracranial pressure assessment. Rather, our aim was to investigate whether mannitol administration could attenuate the increase in a surrogate marker of intracranial pressure during prolonged laparoscopic surgery in the Trendelenburg position.

Once again, we sincerely thank the authors for their constructive comments and for contributing to the ongoing scientific discussion regarding perioperative ONSD assessment.

## AI assistance disclosure

The authors declare that no generative artificial intelligence or AI-assisted technologies were used in the preparation of this manuscript.

## Data availability statement

No new data were created or analyzed in this article. Data sharing is not applicable to this article.

## Authors’ contributions

George Pereira Barreto contributed to the study conception, conducted the study execution, and participated in manuscript drafting. Wallace Andrino da Silva led the study, developed the original idea, coordinated all phases of the project, and was responsible for writing and revising the manuscript.

## Funding

The authors received no financial support for this Letter.

## Conflicts of interest

The authors declare no conflicts of interest.
